# Hydrogel-load exosomes derived from dendritic cells improve cardiac function via Treg cells and the polarization of macrophages following myocardial infarction

**DOI:** 10.1186/s12951-021-01016-x

**Published:** 2021-09-08

**Authors:** Youming Zhang, Zichun Cai, Yunli Shen, Qizheng Lu, Wei Gao, Xin Zhong, Kang Yao, Jie Yuan, Haibo Liu

**Affiliations:** 1 Department of Cardiology, QingPu Branch of Zhongshan Hospital, Fudan University, Shanghai, 201700 People’s Republic of China; 2 Department of Cardiology, Shanghai East Hospital, School of Medicine, Tongji University, Shanghai, 200092 People’s Republic of China; 3 Department of Cardiology, Shanghai East Hospital of Clinical Medical College, Nanjing Medical University, Nanjing, 211166 People’s Republic of China; 4 Shanghai Institute of Cardiovascular Diseases, Zhongshan Hospital, Fudan University, Shanghai, 200032 People’s Republic of China

**Keywords:** Myocardial infarction, Dendritic cell, Exosomes, Alginate hydrogel, Regulatory T cells, Macrophage

## Abstract

**Backgroud:**

Myocardial infarction (MI) is one of the leading causes of global death. Dendritic cell-derived exosomes (DEXs) provide us with the possibility of improving cardiac function after MI but are limited by low retention times and short-lived therapeutic effects. In this study, we developed a novel drug delivery system incorporating alginate hydrogel that continuously releases DEXs and investigated the mechanisms underlying the action of DEXs in the improvement of cardiac function after MI.

**Results:**

We incorporated DEXs with alginate hydrogel (DEXs-Gel) and investigated controlled released ability and rheology, and found that DEXs-Gel release DEXs in a sustainable mammer and prolonged the retention time of DEXs but had no detrimental effects on the migration in vivo. Then DEXs-Gel was applicated in the MI model mice, we found that DEXs-Gel siginificantly enhanced the therapeutic effects of DEXs with regards to improving cardiac function after MI. Flow cytometry and immunofluorescence staining revealed that DEXs significantly upregulated the infiltration of Treg cells and M2 macrophages into the border zoom after MI, and DEXs activated regulatory T (Treg) cells and shifted macrophages to reparative M2 macrophages, both in vitro and in vivo.

**Conclusion:**

Our novel delivery method provides an innovative tool for enhancing the therapeutic effects of DEXs after MI. Further analysis revealed that DEXs exert effect by activating Treg cells and by modifying the polarization of macrophages.

**Graphic Abstract:**

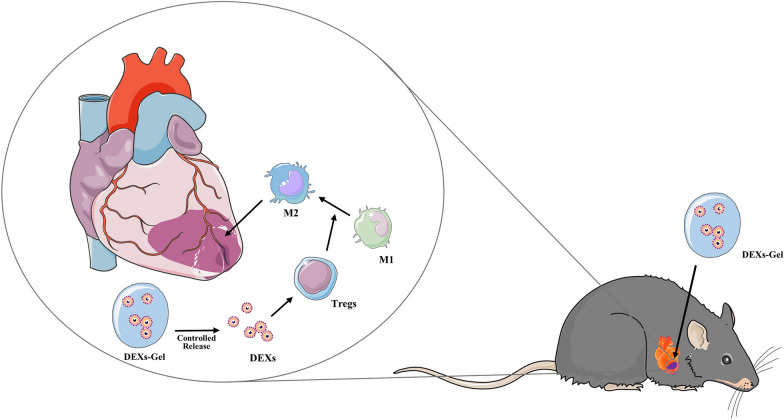

## Introduction

Worldwide, myocardial infarction (MI) is still the primary cause of cardiovascular mortality and disability [1]. The development of clinical treatments, particularly percutaneous coronary intervention and coronary artery bypass grafting, has helped to reduce the mortality associated with MI; however, we do not yet possess the ability to repair and reconstruct damaged myocardium [2]. The progression of MI often leads to adverse ventricular remodeling, including late myocardial fibrosis and irreversible ventricular dilatation; ultimately, these conditions may eventually cause heart failure and death [3, 4].

The immune system plays a central role in both the inflammatory phase and reparative phase after MI [5, 6]; both immune cells and secreted exosomes are known to take part in the reparative phase and act via a paracrine mechanism. Exosomes (EXs) are a type of extracellular vesicle that are secreted by cells and contain proteins, DNA, and RNA [7]. Over recent years, it has become increasingly clear that exosomes from various cell types engage with the immune system as messengers of intercellular communication [7–9]. Dendritic cell (DC)-derived exosomes (DEXs) are known to participate in antigen presentation [10], immune activation, and inhibition [11]. In a previous study we found that DEXs could improve cardiac function via CD4^+^T cells [12]. However, we, and other researchers [13, 14], also found that the intravenous administration of EXs was associated with a short half-life due to clearance by mononuclear phagocytes and accumulation in the liver and spleen; furthermore, the therapeutic effect was significantly limited by low retention rates. However, the simple delivery of a greater number of EXs did not prolong retention times; rather, this generated a range of additional problems [15]. Therefore, there is a clear need to develop a new approach to improve the retention time of DEXs.

Alginate hydrogel (gel) has been widely applied in the fields of tissue engineering and regenerative medicine due to good biocompatibility, the fact that the gelation process is mild, and because its mechanical properties are similar to the extracellular matrix (ECM) [16]. Gel can also provide physical support and increase scar thickness in damaged myocardium when injected into the infarct area after MI [17]. However, whether alginate hydrogel could help to improve cardiac function after MI remains controversial [18–20]. In a recent study, Lv et al. used alginate hydrogel as a carrier for bioactive substances. These authors injected a gel that was loaded with bone marrow mesenchymal stem cell (MSC)-derived small extracellular vesicles (sEVs) into the MI infarct area and found that the gel could prolong the retention of sEVs and enhance the therapeutic effect of sEVs with regards to cardiac function after MI [20]. Thus, we hypothesized that DEXs loaded in gel (DEXs-Gel) might create an efficient drug delivery system to prolong the retention of DEXs. In a previous study, we demonstrated that DEXs improved cardiac function by migrating into the lymphatic system (such as the spleen or lymph nodes) and by activating CD4^+^T cells[12]; however, whether the gel affects the migration of DEXs and whether DEXs-Gel could enhance the therapeutic effects of DEXs remain unknown.

Regulatory T (Treg) cells are a subset of CD4^+^T cells and play a dominant role in the control of self-reactive T cells and the maintenance of immunological self-tolerance [21]. An exaggerated or persistent inflammatory activation after MI leads to maladaptive healing and subsequent remodeling of the left ventricle [22]. Recent studies also showed that Treg cells play a beneficial role in wound healing after MI by modulating the differentiation of monocytes and macrophages [22, 23]. In addition, Choo et al. [24]. showed that the administration of MI mice with dendritic cells (DCs) primed with infarct lysate, or the serum of mice with MI, induced the systemic activation of Treg cells, thus eliciting an earlier macrophage subset shift from inflammatory M1 to reparative M2 macrophages. These alterations in the immune environment within the infarcted heart resulted in better wound remodeling, preserved left ventricle (LV) systolic function after myocardial tissue damage, and improved survival [24]. DEXs contains substances secreted from DCs that may act as intercellular messengers for immune cells [7–9]; they may also participate in antigen presentation [10], immune activation and inhibition [11] as DCs. It is also possible that DEXs could activate Treg cells and lead to an earlier shift in the macrophage subset from inflammatory M1 to reparative M2 macrophages as DCs.

In this study, we developed an injectable alginate-based composite DEXs-Gel in an attempt to enhance the in vivo retention of DEXs and improve its therapeutic effects after MI. We also investigated whether DEXs-Gel could sustainably release DEXs in vitro and used a small animal fluorescence imaging system to compare the retention and migration of DEXs in vivo between the injection of DEXs-Gel and DEXs alone. We also compared the two experimental groups with regards to cardiac function, angiogenesis, myocardium apoptosis, and immune cell activation. Finally, we explored the mechanisms underlying the beneficial effects of DEXs on cardiac function after MI and the superiority of DEXs-Gel in the treatment of MI.

## Materials and methods

### Animals

Wild‑type mice (C57BL/6; all males) were purchased from the Shanghai Laboratory Animal Center, China. Male, 6‑week‑old C57BL/6 mice, weighing 16–20 g, were used for the culture of bone marrow-dendritic cells (BMDCs) and bone marrow-derived macrophages (BMDMs). In addition, 8‑week‑old male C57BL/6 mice, weighing 22–28 g, were used to construct the MI models and were subjected to different injections. All mice were housed under pathogen‑free conditions in a standard laboratory with a controlled room temperature of 22 ± 1 °C, a humidity of 65–70%, and a 12:12‑h light‑dark cycle, with free access to food and water. Animal care and treatment complied with the standards approved by the Institutional Review Board of Zhongshan Hospital of Fudan University and the Shanghai Institutes of Biological Sciences‑CAS (Reference: A5894‑01).

### Cell isolation and culture

BMDCs were isolated from C57BL/6 mice and cultured with a non‑serum medium to eliminate the interference of exosomes caused by fetal bovine serum (FBS), as described previously [12]. In brief, the mice were sacrificed by cervical dislocation and the femurs were isolated. The bone marrow cells were then washed out from the femurs and cultured in Roswell Park Memorial Institute (RPMI) 1640 (Gibco, USA) supplemented with 10% FBS, Life Technologies, USA) and 1% Penicillin/Streptomycin (Gibco, USA) containing 10 ng/ml of granulocyte‑macrophage colony‑stimulating factor and 1 ng/ml of IL‑4 (both from R&D Systems, USA). The medium was changed every other day. On day 7, the BMDCs were collected and treated with the supernatants of necrotic HL‑1 cells for 24 h and then cultured with a non‑serum medium to eliminate the interfering effect of FBS on exosomes.

BMDMs were isolated from C57BL/6 mice, as described above. Then, cells were cultured in RPMI 1640 (Gibco, USA) supplemented with 10% FBS, Life Technologies, USA) and 1% Penicillin/Streptomycin (Gibco, USA) containing 10 ng/ml of macrophage colony‑stimulating factor (R&D Systems, USA). The medium was changed every other day. On day 7, the BMDCs were collected and used for subsequent experiments.

HL-1 cells and mouse thymic regulatory T (Treg) cells were purchased from Bluefbio (Shanghai) Biotechnology Development Co., Ltd and cultured in Dulbecco’s modified Eagle’s medium (DMEM, Gibco) supplemented with 10% FBS (Life Technologies, USA) and 1% Penicillin/Streptomycin (Gibco, USA).

All experiments were performed under sterile conditions and the cultures were maintained at 37 °C in 5% CO_2_ and 95% humidity.

### Simulation of the post‑MI cardiomyocyte microenvironment in vitro

Three types of samples were used to mimic the MI microenvironment: (i) the supernatants of necrotic HL‑1 cells; (ii) the supernatants of hypoxic primary cardiomyocytes; and (iii) the supernatants of infarcted mouse myocardium; the acquisition of these samples was described in our previous study [12]. Data from our previous study suggested that treatment with the supernatants of necrotic HL‑1 cells or hypoxic primary cardiomyocytes in vitro activates DCs in mice in a similar manner to MI. In the present study, we used the supernatants of necrotic HL‑1 cells to mimic the MI microenvironment. In brief, we collected 100 µl of supernatant from necrotic HL‑1 cells. Next, we removed cell membrane particles by centrifugation at 1500*g* at room temperature for 30 min. The purified supernatant of HL-1 cells was then incubated with 1 × 10^6^ BMDCs for 24 h. All experiments were performed under sterile conditions.

### Isolation and characterization of DEXs

In this study, we used the ExoQuick‑Tc™ Exosome Precipitation Solution (System Biosciences Inc.) to isolate DEXs; this system was used in accordance with the manufacturer’s instructions. In brief, a 1/5 volume of ExoQuick‑TC™ Exosome Precipitation Solution was added to 10 ml of supernatant from the DCs and then refrigerated overnight at 4 °C. Then, the samples were centrifuged at 1500*g* at room temperature for 30 min and the supernatant was removed. DEXs were then re‑suspended in 1 ml of PBS (Hyclone; Cytiva) and used for subsequent experiments. Western blotting was then used to identify surface makers on the DEXs, including CD63 (ab193349, Abcam, USA) and Alix (ab275377, Abcam, USA). An H7500 transmission electron microscope (Hitachi, Japan) was then used to determine particle morphology. The particle size distributions of isolated DEXs suspended in PBS was also determined using a Nanosight NS300 (Malvern, UK).

### Cell experimental protocol

Macrophages were treated with 100 ng/ml of lipopolysaccharide (LPS, Sigma-Aldrich) or 10 ng/ml of IL-4 and IL-13 (PeproTech, USA) for 48 h and used as M1- or M2-positive controls. DEXs (40 μg/ml) or Treg cells (1 × 10^6^ cells/ml) were incubated with 2 × 10^6^ macrophages for 48 h and used as experimental groups. Untreated cells were used as negative controls. After 48 h of culture, we collected cells from each group for flow cytometry.

### Flow cytometry analysis

Samples were incubated with an Fc receptor blocker (CD16/32, BD Bioscience) to reduce nonspecific antibody binding. Freshly isolated samples were resuspended in staining buffer (R&D Systems) and stained with F4/80‐FITC (eBioscience) for 30 min at 4 °C. For intracellular staining, we used the Intracellular Fixation and Permeabilization Kit (BD Bioscience); this was used in accordance with the manufacturer’s instructions. Cells were then washed and stained with iNOS-APC (eBioscience) and CD206-PE (eBioscience). Flow cytometry was performed using a FACS Aria flow cytometer (BD Bioscience) and data were analyzed with FlowJo V10.6.2 software (TreeStar, Ashland, OR).

### Incorporating DEXs with alginate hydrogel

We mixed 2% (wt/vol) sodium alginate and 1.5% (wt/vol) calcium alginate (both from Sigma) at a volume ratio of 1:1 to form the alginate hydrogel. DEXs (30 µg DEXs in 15 μl of gel) were then added into the alginate solution which was then stirred and mixed to form alginate hydrogel incorporating DEXs. A MARS60 (HAAKE, Germany) rheometer was used to evaluate rheological behavior. Finally, the samples were freeze dried and microstructure was observed with a Gemini 300 transmission electron microscope (ZEISS, Germany).

### Assessing the release ability of DEXs-Gel

For release studies, 100 μl of alginate hydrogel incorporating 80 μg of DEXs were incubated in 200 μl of PBS (Hyclone; Cytiva) at 37 °C in a 5% CO_2_ atmosphere. The supernatant was removed and fresh PBS (Hyclone; Cytiva) was added every 2 days. The number of DEXs released into the medium was then determined by the Enhanced BCA Protein Assay Kit (P0010, Beyotime, Biotechnology, Shanghai, China).

### Induction and treatment of a mouse model of MI

Coronary artery ligation was used to induce a mouse model of MI. In brief, mice were anesthetized by 2% isoflurane inhalation (RWD Life Science, China) with an isoflurane delivery system (Harvard Bioscience, Inc.) and then ventilated with a rodent ventilator (Harvard Bioscience, Inc.). A small incision was made on the skin of the left chest to allow us to dissect and retract the major and minor pectoral muscles. Next, we opened the chest, exposed the heart, and used 8–0 silk sutures to ligate the left coronary artery (LCA). Successful infarction was determined by observing the blanching of the myocardium in the affected area. Mice undergoing the same surgical procedure, but without LCA ligation, were used as a sham group. After successfully constructing the MI model, we injected 15 µl DEXs (30 µg DEXs in 15 µl PBS), Gel, or DEXs-Gel (containing 30 µg of DEXs), into the myocardium of the left ventricle at three sites (two sites at the border and one site at the infarct area). Finally, we evacuated remaining air, sutured the skin, placed the mice back to their cages, and waited for them to come round from the anesthesia.

### DEXs labeling and fluorescence imaging

DEXs suspended in PBS (Hyclone; Cytiva) were labeled with a 1:400 dilution of DiR (Thermo Fisher Scientific) for 30 min at 37 °C. Excess dye was then removed and the DEXs purified using exosome spin columns (MW 3000; Thermo Fisher Scientific). Near-IR fluorescence images were then acquired using the IVIS Spectrum System (PerkinElmer). We used this system to compare the maximal fluorescence intensity of the body and main organs between the DEXs and DEXs-Gel groups.

### Echocardiography

In vivo cardiac function was determined by echocardiography (Vevo 2100, VisualSonics, Toronto, Canada) on day 7 and day 14 post-surgery. Mice were anesthetized with 2% isoflurane (RWD Life Science, China) and oxygen, and two-dimensional echocardiographic views of the left ventricular long axis were obtained through the anterior and posterior LV walls at the level of the papillary muscle tips below the mitral valve. Then, we used Vevo 2100 workstation software to calculate the left ventricular ejection fraction (LVEF), left ventricular fractional shortening (LVFS), left ventricular end-diastolic diameter (LVIDD), and left ventricular end-systolic diameter (LVIDS). All echocardiograms were evaluated in a blinded manner.

### Histology

Mice were sacrificed by cervical dislocation on day 28. Then, we used Masson’s trichrome staining to determine infarct size and the thickness of the wall in the infarct zone. In brief, heart samples were fixed with 4% paraformaldehyde at room temperature for 24–48 h. Then, we embedded samples of heart tissue in paraffin and prepared tissue sections (5 µm in thickness); these were then dewaxed in xylene (Sangon Biotech Co., Ltd.) and rehydrated using ethanol. Finally, sections were stained with Masson’s trichrome reagent and sealed with neutral gum. After observation with an optical microscope, the infarct area was calculated by the sum of the endocardial and epicardial length of the infarct zone divided by the total length of the endocardial and epicardial left ventricle. In addition, the thickness of the wall in the infarct area was averaged from three to five measurements of scar thickness using Image J V1.53e software.

### Immunofluorescence staining

We also harvested samples of heart tissue for paraffin-embedding and sectioning on days 3, 5, and 14. The sections were dewaxed, rehydrated and antigen retrieval was performed by boiling the sections in 0.01 M citrate buffer (Sangon Biotech Co., Ltd. Shanghai, China). Then, the sections were incubated overnight at 4 °C with rabbit anti- iNOS (ab3523, Abcam, USA), rabbit anti-CD206 (ab64693, Abcam, USA), and rabbit anti-Foxp3 (ab75763, Abcam, USA). Rabbit anti-CD31 (GB11063, Servicebio Technology Co., Ltd. Wuhan. China) was used to investigate angiogenesis. Cardiac Troponin T Rabbit pAb (GB11364, Servicebio Technology Co., Ltd. Wuhan. China) was used to determine the extent of viable myocardium in the infarct area. Next, the sections were incubated with corresponding fluorescent dye-conjugated secondary antibodies (GB25303; GB21303, Servicebio Technology Co., Ltd. Wuhan. China) and nuclei were counterstained with DAPI (G1012, Servicebio Technology Co., Ltd. Wuhan. China) in accordance with the manufacturer’s instruction. We also used a TUNEL kit (G1501, Servicebio Technology Co., Ltd. Wuhan. China) to evaluate cell apoptosis. The relative proportions of cells that were positive for iNOS, CD206, Foxp3, and TUNEL, were then determined from the total number of cells; four hearts were evaluated per group. We also counted the number of CD31-positive blood vessels in four hearts per group. All evaluations were performed in the border zones and analyzed with Image J V1.53e software.

### Statistical analysis

Results are shown as mean ± standard deviation (SD). Statistical analyses of differences between two groups were performed using the unpaired *t*-test while differences between three or more groups were performed by one-way analysis of variance (ANOVA) followed by post-hoc Tukey’s multiple tests. Statistical tests were performed with Prism GraphPad Prism V8.0.1 Software (GraphPad Prism Software Inc.). p < 0.05 was considered to be statistically significant.

## Results

### Characterization of DCs and DEXs

**Fig. 1:**
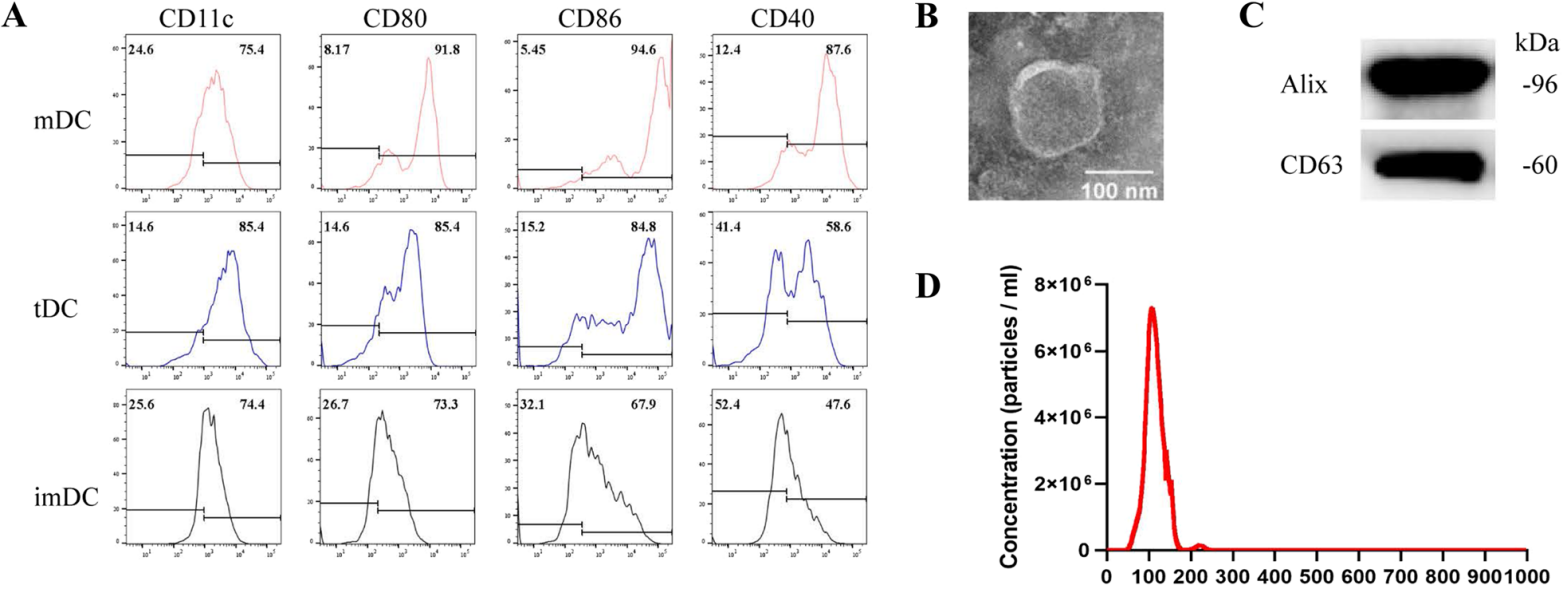
Characterization of DCs and DEXs. **A** Flow cytometry analysis of DC subsets (mDC, tDC, and imDC). Representative data are presented as histograms. **B** Representative transmission electron microscopy (TEM) image of DEXs. **C** Western blot analysis of ALIX and CD63 in DEXs. **D** Nanoparticle tracking analysis of dendritic cells-derived exosomes (DEXs); the mode diameter was 106.5 nm

We used the supernatants of necrotic HL-1 cells to mimic the MI microenvironment. Cultured BMDCs were either unstimulated (imDCs) or stimulated with the supernatants of necrotic HL-1 cells (tDCs), or LPS (mDCs), for 24 h and then analyzed by flow cytometry. Figure 1A shows that supernatants from necrotic HL-1 cells upregulated the expression of several markers of DC maturation (CD40, CD80, and CD86), as demonstrated in a previous study [24].

Next, we used the ExoQuick‑Tc™ Exosome Precipitation Solutio to isolate DEXs from the culture medium of DCs. The ultrastructure of the DEXs were demonstrated by transmission electron microscopy (TEM) (Fig. 1B) and particle size distributions were determined via nanoparticle tracking analysis (NTA); the mode diameter was 106.5 nm (Fig. 1D). We also used western blotting to demonstrate the expression of exosomal markers (CD63 and Alix; Fig. 1C).

### Characterization of the Gel and DEXs-Gel

**Fig. 2:**
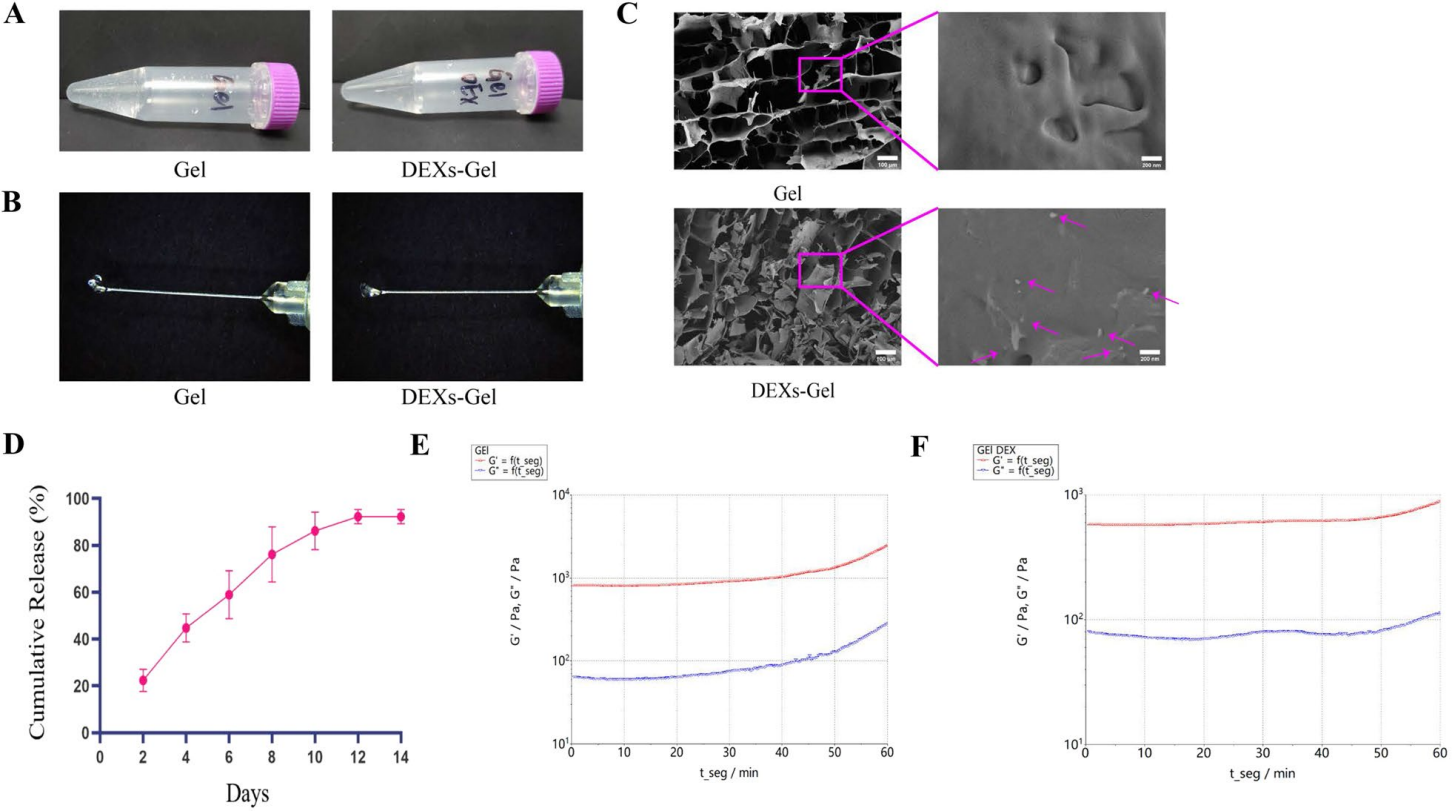
Characterization of the Gel and DEXs-Gel. **A** General appearance of Gel and DEXs-Gel. **B** The injectability of Gel and DEXs-Gel were examined via a 29G needle. **C** Representative scanning electron microscopy images of Gel and DEXs-Gel. **D** The curve of DEXs-Gel controlled release ability. **E** and **F** Rheological behavior of Gel and DEXs-Gel. G′, storage modulus. G″, loss modulus.

The Gel and DEXs-Gel (Fig. 2A) were both achromatous and existed in a non-liquid form. Next, we evaluated the injectability and viscosity of the hydrogel with a 29G needle (Fig. 2B). Next, the microstructure of the Gel and DEXs-Gel were captured by scanning electron microscopy (Fig. 2C). Then, the rheological properties of the Gel and DEXs-Gel were examined (Fig. 2E, F); we found that their storage modulus (G′) lay between 400 and 1800 Pa; this is considered to represent an appropriate G′ for cardiac tissue engineering [25]. Finally, we determined the controlled release ability of the DEXs-Gel (Fig. 2D); analysis showed that DEXs-Gel could release DEXs in a controlled manner for 10–12 days in vitro*.*

### DEXs-Gel released DEXs in a sustainable manner and prolonged the retention of DEXs in vivo

**Fig. 3:**
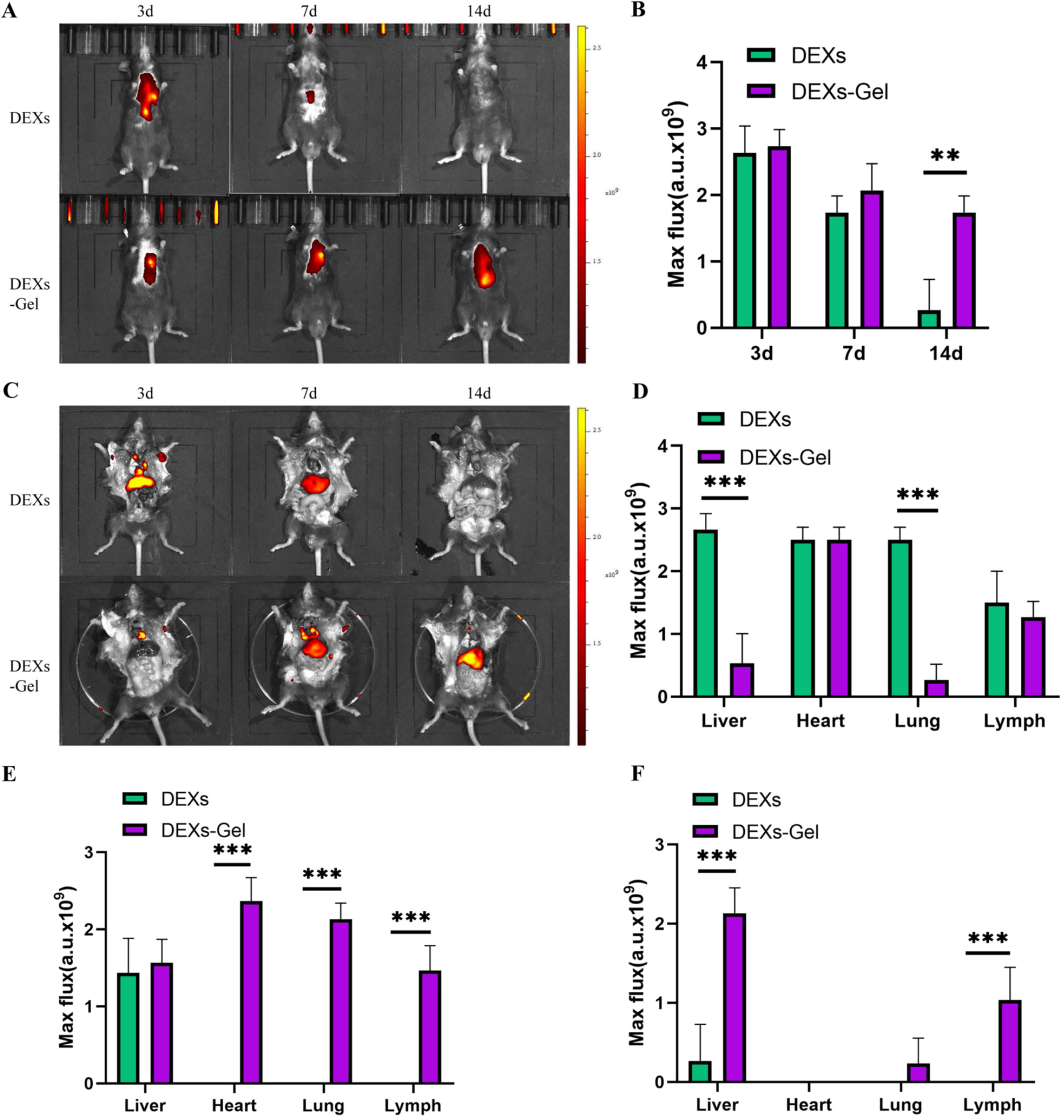
DEXs-Gel prolonged the retention time of DEXs in vivo. **A** and **C** Near-IR fluorescence images of the body and main organs. **B** Analysis of maximum flux (a.u.) of body near-IR fluorescence in different groups. **D**–**F** Analysis of maximum flux (a.u.) of different organs in terms of near-IR fluorescence in different groups on day 3, day 7 and day 14. n = 3, *p < 0.05, **p < 0.01, ***p < 0.001

Next, we determined the release ability of DEXs-Gel by using a BCA Protein Assay Kit to detect the DEXs that had been released into the medium. We found that DEXs-Gel could sustainably release DEXs in vitro (Fig. 2D). For in vivo studies, we labeled DEXs with DIR before incorporating them into alginate hydrogel; then, we injected DEXs alone, or DEXs-Gel, into the infarcted area in MI mice. On days 3, 7 and 14, we acquired near-IR fluorescence images from the body and main organs with a IVIS Spectrum System. The results (Fig. 3A and B) showed that there were fewer remaining DEXs in the DEXs injection group on day 7 compared with the DEXs-Gel injection group. By day 14, DEXs had been eliminated in the DEXs injection group; however, an abundance of DEXs had been retained by the DEXs-Gel injection group. These results indicated that alginate hydrogel prevented the clearance of DEXs and prolonged its retention in vivo. To investigate the distribution of DEXs in vivo, we evaluated fluorescent signals in the heart, lungs, livers, and lymph of each group (Fig. 2C–F). Although we injected DEXs into the heart, we found that they had migrated to the axillary lymph nodes in both groups by day 3. In the DEXs-Gel injection group, we observed DEXs in the inguinal lymph nodes on day 7. These results indicated that DEXs participated in antigen presentation as dendritic cells, as described previously [10]. However, we observed stronger fluorescent signals in the liver in the DEXs injection group when compared with the DEXs-Gel injection group on day 3. This indicated that that more DEXs had been eliminated in the DEXs injection group; the liver is the main organ responsible for the elimination of DEXs [13, 14]. On day 7, few DEXs had been retained in the hearts or lymph nodes of mice in the DEXs injection group; almost all fluorescent signals were in the liver. This information suggested that the majority of the DEXs were not functional in vivo, however, although an abundance of DEXs were observed in the heart and lymph nodes in the DEXs-Gel injection group. On day 14, the DEXs in the DEXs-Gel injection group had entered the clearance stage but had been cleared completely in the DEXs injection group.

### DEXs-Gel enhanced cardiac function after MI

**Fig. 4:**
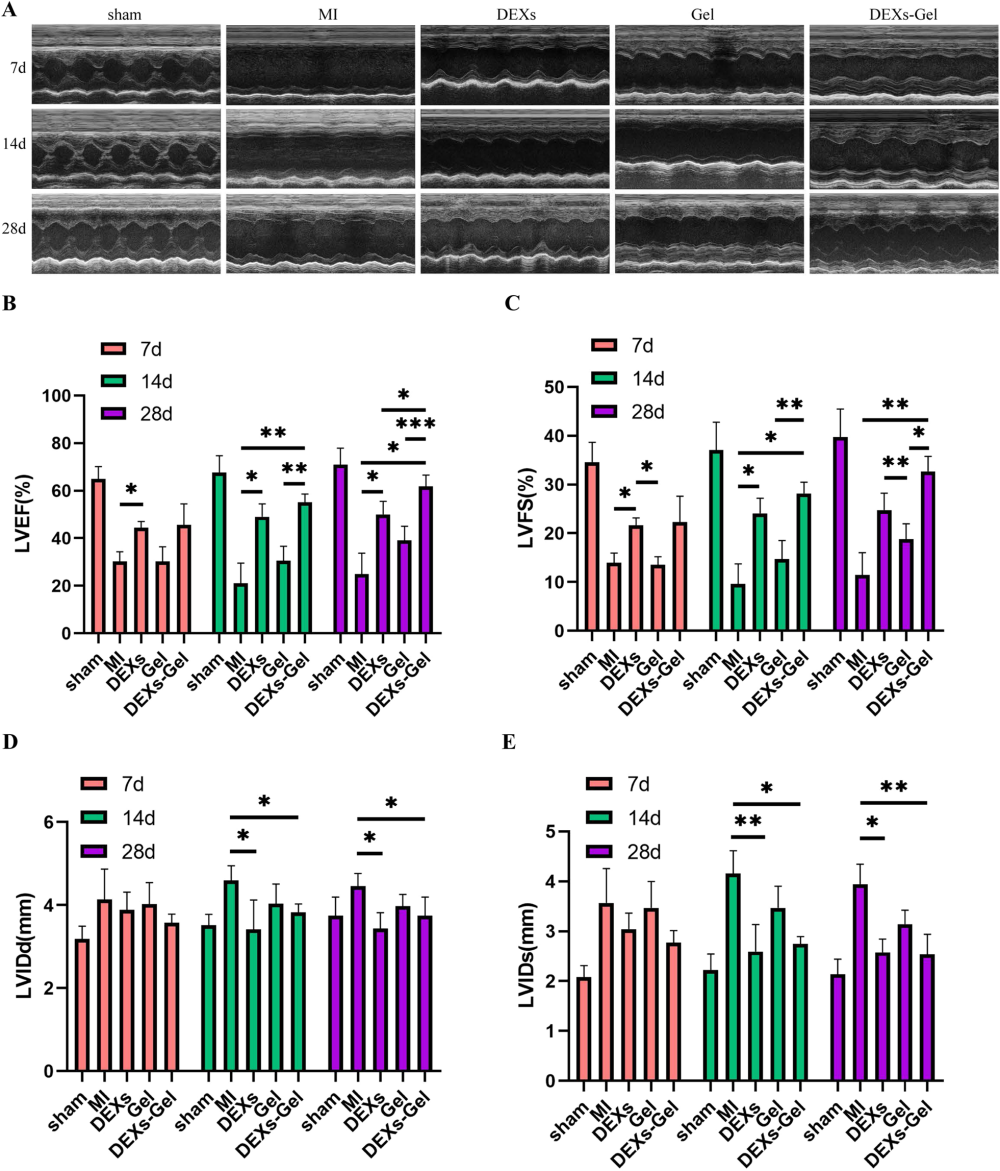
DEXs-Gel enhanced cardiac function after MI. **A** Representative images of echocardiography in different groups. **B**–**E** Analysis of left ventricular ejection fraction (LVEF), left ventricular fraction shortening (LVFS), left ventricular end diastolic diameter (LVIDd), and left ventricular end systolic diameter (LVIDs). n = 5, *p < 0.05, **p < 0.01, ***p < 0.001

We performed echocardiography on days 7, 14 and 28 after surgery. Figure 4A–E show that the DEXs had demonstrated their therapeutic effect by improving cardiac function on day 7; however, the Gel injection did not show any obvious effects with regards to the recovery of cardiac function in mice on day 7, as described previously [18, 19]. However, on day 14, the cardiac function of the MI group was worse than on day 7; mice in the and Gel injection group had maintained their cardiac function and showed distinct advantages compared with the MI group. Of all groups, mice in the DEXs-Gel injection group exhibited the best performance in terms of improved cardiac function, demonstrating almost the same effect as that in the DEXs injection group on day 7, but with significant improvement on day 14. On day 28, the cardiac function of mice in the MI group had become slightly better than on day 14. Mice in the Gel group maintained a similar level of cardiac function as on day 14, thus indicating that Gel injection did not improve cardiac function but could prevent cardiac function from becoming worse. Mice in the DEXs and DEXs-Gel group improved cardiac function; mice in the DEXs-Gel showed better effects than mice in the DEXs group. These results indicated that DEXs-Gel not only prolonged the retention time of DEXs but also improved the therapeutic effect.

### DEXs regulated inflammation via Treg cells and M2 macrophage infiltration

**Fig. 5:**
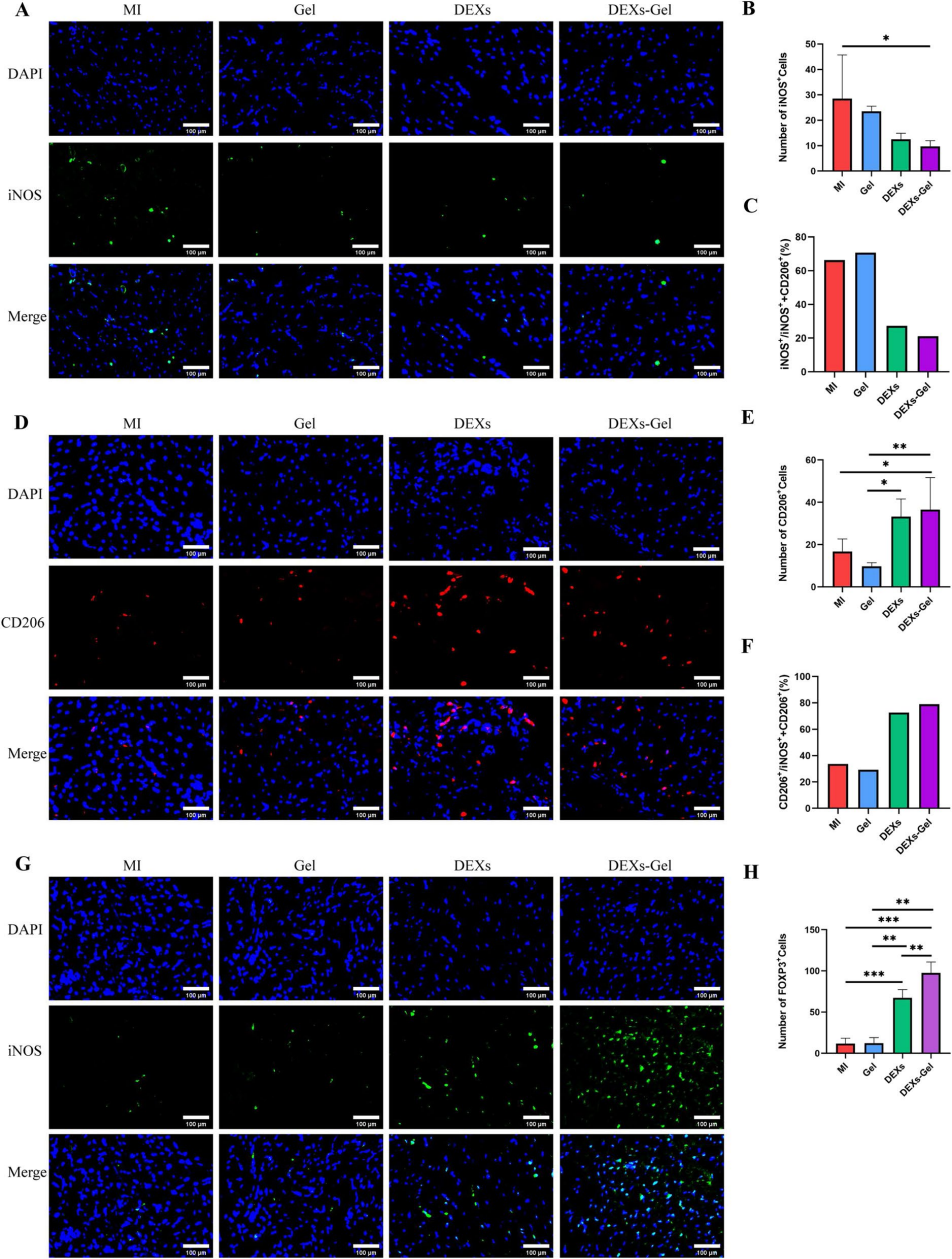
DEXs regulated inflammation via Treg cells and M2 macrophage infiltration. **A** Representative immunofluorescence staining images of iNOS^+^M1 macrophages in the border zone on day 5. **B** Number of iNOS^+^ cells. n = 4, *p < 0.05. **C** Analysis of M1/(M1 + M2) ratio. **D** Representative immunofluorescence staining images of CD206^+^ M2 macrophages in the border zone on day 5. **E** The number of CD206^+^ cells. n = 4, *p < 0.05, **p < 0.01. **F** Analysis of M2/(M1 + M2) ratio. **G** Representative immunofluorescence staining images of Foxp3^+^Treg cells in the border zone on day 5. **H** Analysis of Foxp3^+^ cells. n = 4, *p < 0.05, **p < 0.01, ***p < 0.001

Although the inflammatory cascade plays an important role in the response to cardiac injury, excessive inflammatory reactions are not conducive to heart repair. Evidence from two recent trials showed that the anti-inflammatory effects of colchicine reduced the risk of cardiovascular events in patients with recent myocardial infarction [26, 27]. A study on MI mice models also found that although heart function is enhanced by stem cell therapy after MI, it was not associated with the production of new cardiomyocytes; rather, the enhancement was associated with an acute sterile immune response characterized by macrophages [28]. Collectively, these results emphasized the vital effects of anti-inflammatory therapy after MI. We investigated the therapeutic effect of DEXs and DEXs-Gel in terms of improvements in cardiac function; however, the precise mechanisms involved has yet to be elucidated. Therefore, we detected the immunoregulation status in each group of mice. Considering that pro-inflammatory cells peak on day 3 after MI and anti-inflammatory cells peak on days 5–7 [2, 29], we chose day 5 after surgery to detect immune cell infiltration by immunofluorescence staining. We found that the injection of DEXs reduced the infiltration of iNOS^+^ M1 pro-inflammatory macrophages and increased the infiltration of CD206^+^ M2 anti-inflammatory macrophages and Foxp3^+^ Treg cells into the border zone (Fig. 5A–H). These results confirmed our hypothesis that DEXs could activate Treg cells, thus leading to an earlier shift in the macrophage subset from inflammatory M1 to reparative M2 macrophages as DCs.

### DEXs regulated the polarization of macrophages via Treg cells

**Fig. 6:**
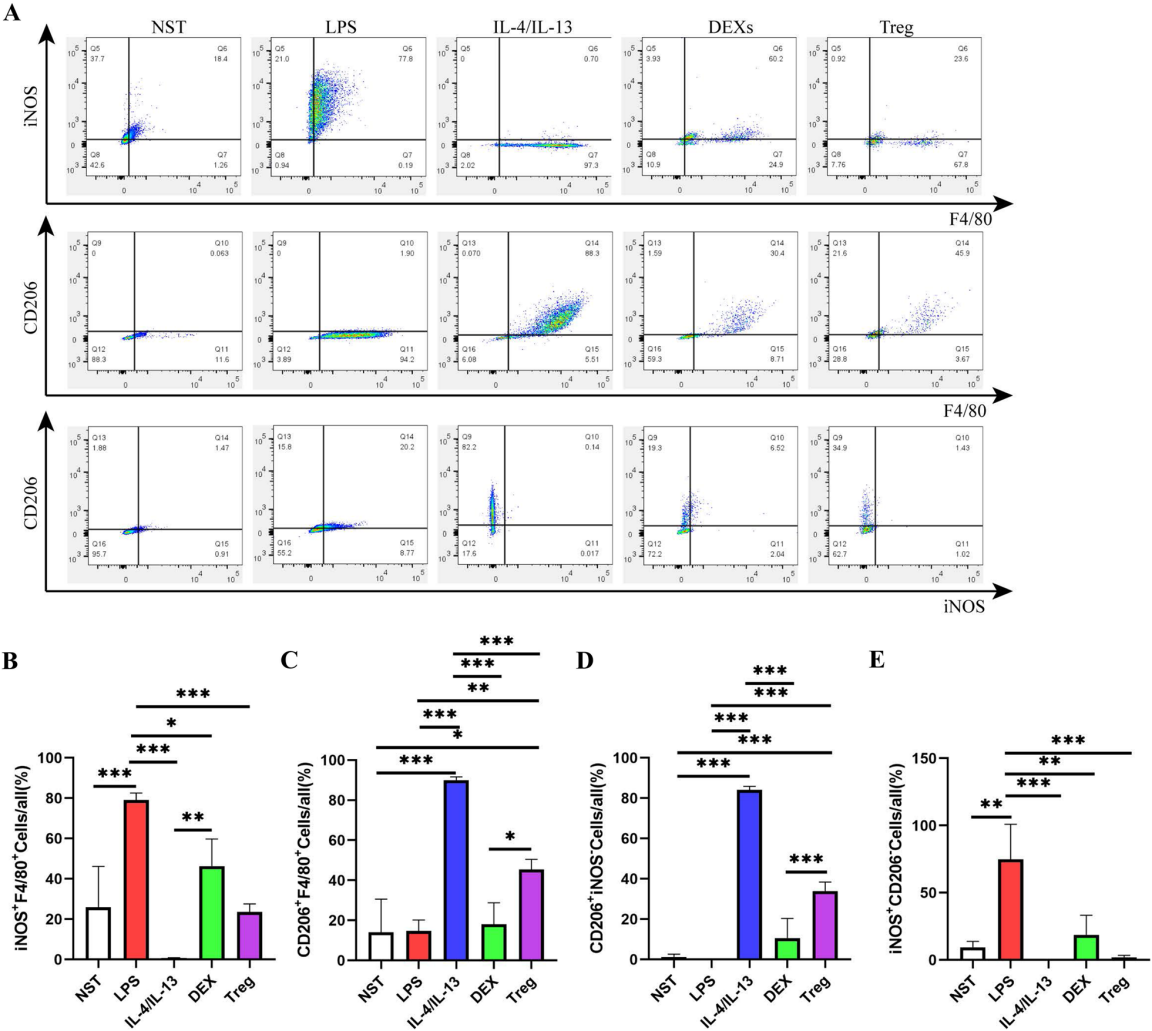
DEXs regulated macrophage polarization via Treg cells. **A** Representative flow cytometry analysis images of different groups. NST, non-treated macrophages; LPS, LPS treated macrophages; IL-4/IL-13, IL-4 and IL-13 treated macrophages; DEX, macrophages co-cultured with DEXs; Treg, macrophages co-cultured with Treg cells. **B**–**E** Analysis of the proportions of iNOS^+^F4/80^+^ cells, CD206^+^F4/80^+^ cells, CD206^+^iNOS^−^ cells, iNOS^+^CD206^−^ cells in different groups. n = 3–4, *p < 0.05, **p < 0.01, ***p < 0.001

We previously found that DEXs activated Treg cells and regulated the polarization of macrophages after MI, although the specific mechanism involved remained unknown. To solve this problem, we co-cultured DEXs, macrophages, and Treg cells, in vitro and determined the ratio of M1/M2 macrophages by flow cytometry. We found that Treg cells could shift macrophages to become M2-like type cells rather than M1-like type cells; however, DEXs stimulated macrophages to express both M1 and M2-like type cells with a greater tendency for the M1-like type than the M2-like type (Fig. 6A–E). Considering that the immunofluorescence staining results showed a significant infiltration of Treg cells and M2 macrophages into the border zone after the injection of DEXs or DEXs-Gel, we hypothesized that DEXs regulate M1/M2 macrophage polarization via Treg cells and not by themselves. Furthermore, near-IR fluorescence images also confirmed that DEXs had migrated into lymph nodes after intramyocardial injection. Thus, we concluded that DEXs had migrated into the lymph nodes and activated Treg cells to regulate the shift of macrophages towards M2-like type cells after intramyocardial injection and thus improved cardiac function post-MI.

### DEXs protected cardiac cells against apoptosis and promoted angiogenesis

**Fig. 7:**
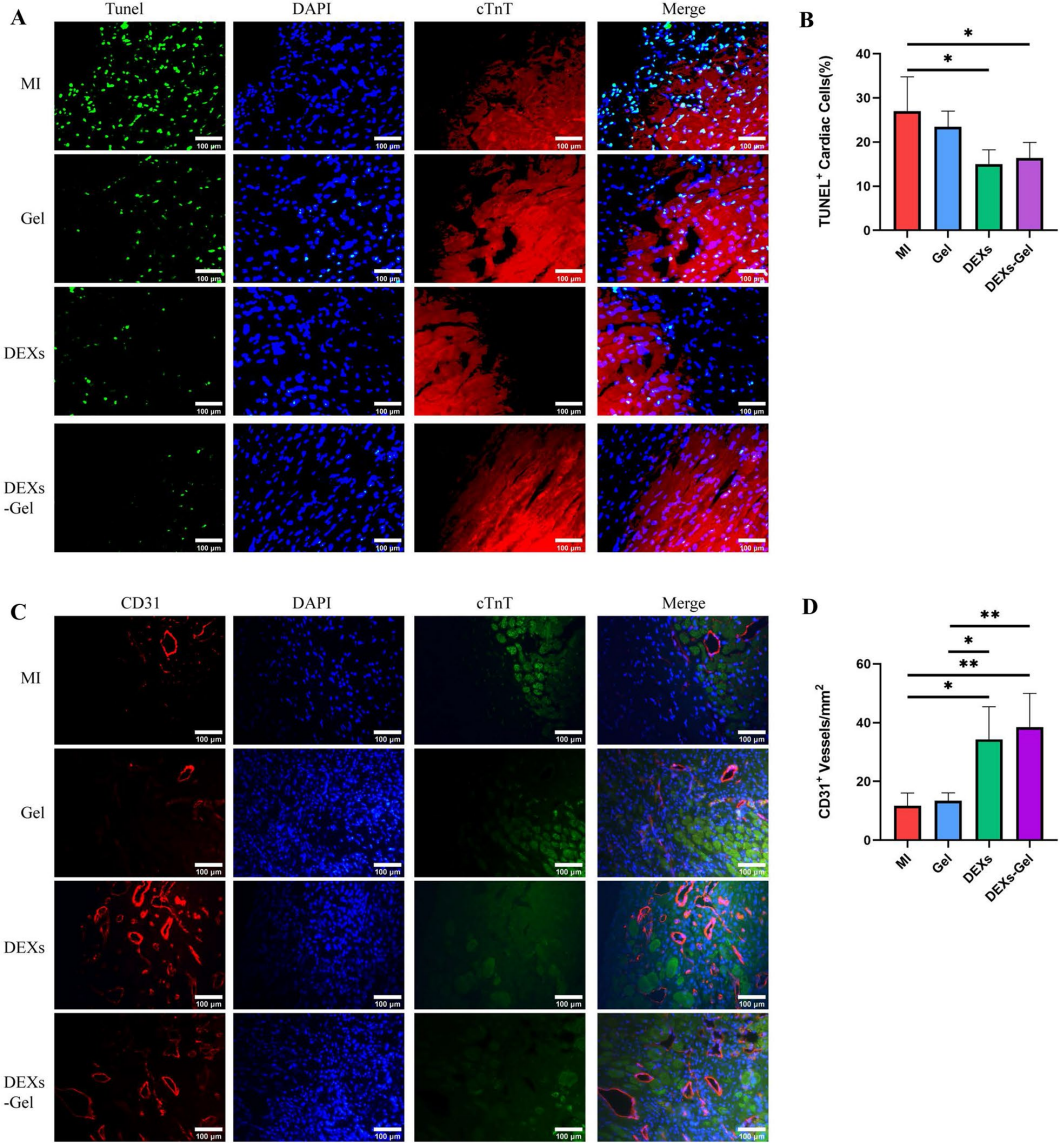
DEXs protected cardiac cells against apoptosis and promote angiogenesis. **A** Representative TUNEL staining images in different groups in the border zone on day 3. **B** Analysis of TUNEL^+^ cardiac cells percentage in different groups. n = 4. **C** Representative CD31 staining images of different groups in the border zone on day 14. **D** Analysis of CD31^+^ vessels/mm^2^. n = 4, *p < 0.05, **p < 0.01, ***p < 0.001

Ischemia and hypoxia, generated by the blocked blood supply to the heart after MI, inevitably results in damage to the cardiac cells. The regeneration of blood vessels is important if we are to rescue ischemic myocardium and avoid the apoptosis of cardiac cells. Therefore, we compared cardiac cell apoptosis via TUNEL staining on day 3 and angiogenesis via CD31 staining on day 14. We found that the proportion of apoptosis cells in the border zoom were markedly lower in the DEXs and DEXs-Gel injection group when compared with the MI and Gel injection group (Fig. 7A and B). Furthermore, we observed new vessels that stained positive for CD31 (Fig. 7C and D) in the DEXs and DEXs-Gel injection group. Furthermore, the DEXs-Gel injection group exhibited the best ability to promote angiogenesis when compared to the other groups. Collectively, these results indicated that DEXs can protect cardiac cells against apoptosis and promote angiogenesis. Data also indicated that DEXs-Gel prolonged the therapeutic effects of DEXs therapeutic effects but did not enhance these effects directly.

### DEXs-Gel increased the thickness of the infarct wall and reduced infarct size

**Fig. 8:**
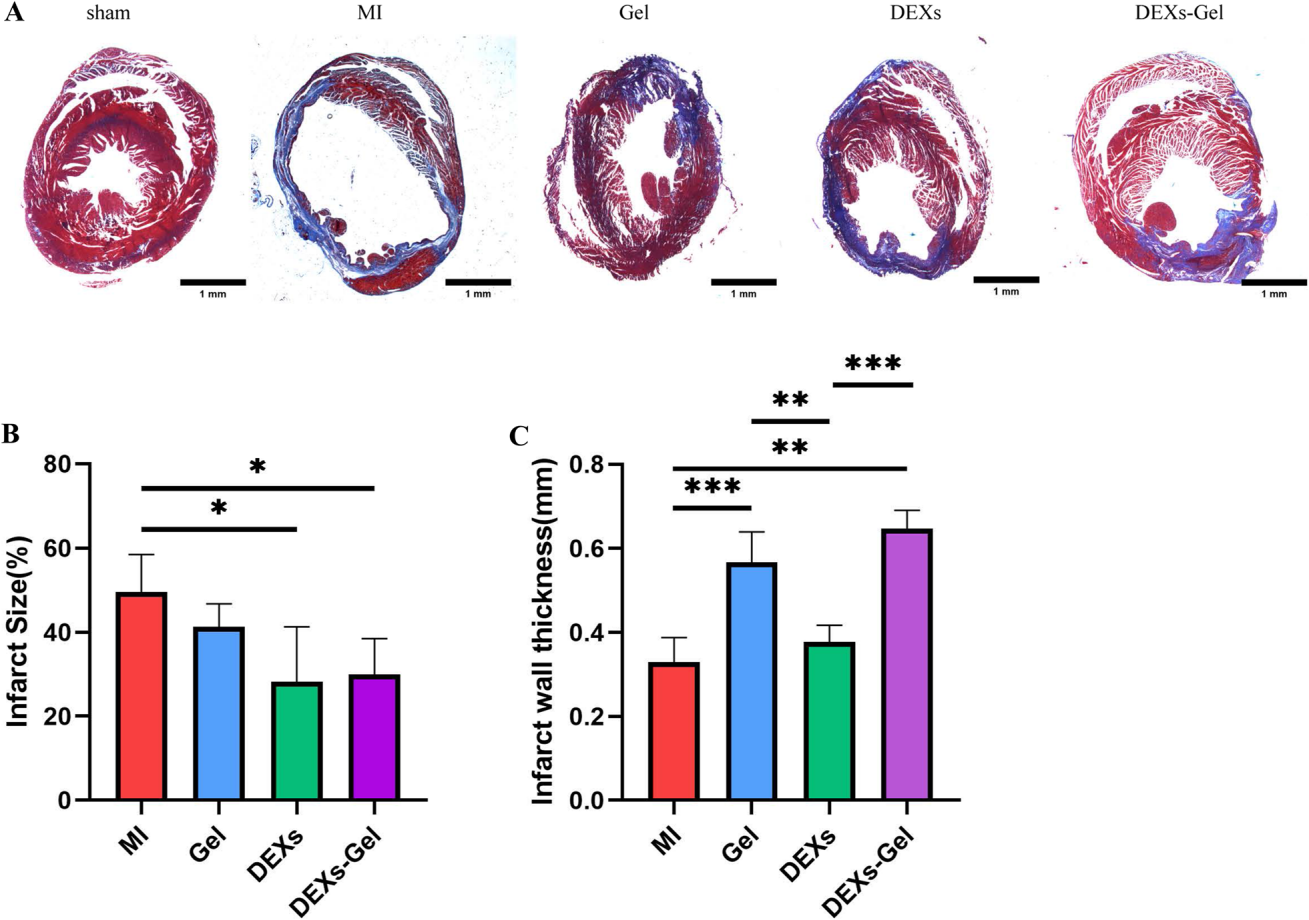
DEXs-Gel increased the infarct wall thickness and reduced infarct size. **A** Representative Masson’s trichrome staining images in different groups. **B** Analysis of infarct size (%) in different groups. n = 4. **C** Analysis of infarct thickness (mm) in different groups. n = 4, *p < 0.05, **p < 0.01, ***p < 0.001

We confirmed that DEXs-Gel could enhance cardiac function via immunoregulation, anti-apoptosis, and by promoting angiogenesis. Next, we investigated these processes by histological analysis. For each group, we performed Masson’s trichrome staining on day 28 after surgery. We found that Gel injection provided physical support and increased the scar thickness in damaged myocardium when injected into the infarcted area after MI (Fig. 8A–C), as previously described [17]. Furthermore, the injection of DEXs reduced the infarct size and rescued more damaged myocardium when compared to the MI and Gel group. DEXs-Gel injection yielded two beneficial effects related to DEXs while Gel injection resulted in better cardiac function improvement when compared to the other groups. These results indicated that DEXs-Gel not only prolonged the retention time of DEXs; they also provided more physical support than DEXs injection and thus enhanced cardiac function after MI, thus suggesting that DEXs-Gel is a better approach for the treatment of MI.

## Discussion

In this study, we demonstrated that DEXs significantly upregulated the infiltration of Treg cells and M2 macrophages into the border zoom after MI, thus indicating that DEXs improved cardiac function via these two types of cells. Furthermore, we performed flow cytometry to identify the specific relationships among DEXs, Treg cells, and macrophages, and found that Treg cells shifted macrophages into M2-like type cells; in contrast, DEXs stimulated macrophages to express both M1 and M2-like type cells but with a greater tendency towards the M1-like type. Collectively, these results indicated that DEXs activated Treg cells, and that the activated Treg cells then regulated the polarization of macrophages and shifted macrophages into an M2-like type to protect cardiac function after MI. To address the low retention rates and short-lived therapeutic effects of DEXs, we used an alginate hydrogel to load DEXs. Previous studies showed that the Gel had good tolerance with a low incidence of adverse events [16], and that it could provide physical support and increase scar thickness in damaged myocardium when injected into the infarcted area after MI [17]. However, these is still controversy with regards to whether a single Gel injection could help improve cardiac function after MI [18–20]. Our results demonstrated that a single Gel injection increased the thickness of the infarcted wall but failed to improve cardiac function after MI. However, this strategy did protect cardiac function from exacerbating over time when compared with the MI group. Considering that the Gel provides physical support to the infarcted area after MI, the combined application of Gel with bioactive factors that have therapeutic effects could provide a much better approach. Previous research showed that the Gel could prolong the retention of sEVs and improve cardiac function after MI [20]. However, prior to this study, it remained unknown whether Gel could affect the migration of DEXs or enhance their therapeutic effects with regards to protecting cardiac function after MI. In the present study, we found that DEXs-Gel prolonged the retention time of DEXs in vivo and had no detrimental effects on the migration of DEXs. We also found that Gel enhanced therapeutic effects with regards to cardiac function after MI, thus indicating that DEXs-Gel is a superior approach for delivering DEXs.

MI and subsequent adverse ventricular remodeling are still a major health concern worldwide [1]. Over recent decades, researchers have gradually realized the important role of inflammatory reactions after MI. Excessive inflammation leads to adverse ventricular remodeling and become harmful with regards to the recovery of cardiac function. Evidence from two recent trials also showed that the anti-inflammatory effects of colchicine reduce the risk of cardiovascular events in patients with recent myocardial infarction [26, 27]. An investigation of MI mice models also showed that stem cell therapy can enhance heart function after MI, these effects were not associated with the production of new cardiomyocytes; rather, this enhancement occurred via an acute sterile immune response characterized by macrophages [28]. These results emphasized that the appropriate application of anti-inflammatory therapy after MI could be of benefit to patients with MI. However, not all applications of anti-inflammatory drugs can attenuate injury [30], thus emphasizing the complex nature of inflammatory reactions after MI and indicating that an effective therapy should not only reduce the length and damage of the inflammatory response but also promote the activation of reparative pathways. As a novel non-cell therapy, EXs have been reported to play an important role in myocardial healing and cardiac remodeling after MI [31–33]. However, the majority of previous studies focused on the role of EXs secreted by stem cells, cardiomyocytes, or fibroblasts [32, 33]. In comparison, the potential role of EXs from immune cells has not received significant levels of attention even though they make a significant contribution to the repair of myocardial injury after MI [12, 34]. As EXs are secreted by DCs, our previous studies demonstrated that DEXs could improve cardiac function by activating CD4^+^ T cells [12] and promote angiogenesis by exosomal miR-494-3p [34]. DEXs and miR-494-3p are both potential targets for non-cell therapies. Some recent studies have shown that the use of hydrogel as a carrier for microRNA is a safe and effective interventional strategy to delay and reverse pathological progression [35, 36]. However, we and some other researchers [37], found that the inflammation following MI is associated with the apoptosis of myocytes, angiogenesis and fibrosis. Considering the vital role of inflammation after MI, we chose DEXs as our target for investigation. Therapy involving EXs still faces a major challenge with regards to a short half-life that imposes a significant limitation on therapeutic effects [13, 14]. Thus, we combined DEXs with Gel and developed a novel delivery system for DEXs that did not affect DEXs migration and enhanced their therapeutic effects after MI. DEXs-Gel is easy to produce and has no detrimental effects when injected into the myocardium. This product cannot only provide physical support to the infarcted myocardium; it may also provide benefits with regards to immunoregulation, anti-apoptosis, and angiogenesis. DEXs-Gel overcomes the short-therapeutic effects of therapy involving DEXs and provides a long-term favorable consequence to MI by combining the two advantages of Gel and DEXs with regards to the protection of cardiac function after MI. Furthermore, we clarified the mechanism responsible for the migration of DEXs into the lymph nodes and the activation of Treg cells (a subset of CD4^+^T cells) after MI. We found that Treg cells regulate the polarization of macrophages in post-MI hearts and shifts macrophages towards M2-like type cells in order to improve cardiac function.

However, we still need to investigate how DEXs activate Treg cells and how Treg cells regulate the polarization of macrophages. A recent study revealed that Treg cells could stimulate macrophages producing IL-10 by secreting IL-13 [23]. Considering the secretion of IL-10 is one of the characteristic features of M2-like type macrophages [38], Treg cells may regulate macrophage polarization via IL-13. However, whether DEXs simulate CD4^+^ T cells to differentiate into Treg cells or activate Treg cells in lymph nodes directly, still needs further investigation. Moreover, our study of DEXs-Gel was carried out in a mouse model. In our future research, we will utilize large animal, such as the pig, to investigate the potential of this technique for clinical translation.

## Conclusion

In this study, we confirmed that DEXs-Gel prolonged the retention time of DEXs and had no detrimental effects on the migration of DEXs, therefore enhancing the therapeutic effects of DEXs with regards to improving cardiac function after MI. Compared with single Gel or DEXs injection, the injection of DEXs-Gel had better consequences on immunoregulation, anti-apoptosis, the promotion of angiogenesis, and also increased the thickness of the infarcted wall. We also investigated the mechanisms by which DEXs improved cardiac function and found that DEXs activated Treg cells which then regulated macrophage polarization and shifted macrophages towards M2-like type cells. Our study developed a novel strategy for delivering DEXs and provided new insight into the use of immunotherapy to treat MI.

## Data Availability

The datasets used and/or analyzed during the current study are available from the corresponding author on reasonable request.

## Abbreviations

MI: Myocardial infarction
DCs: Dendritic cells
BMDCs: Bone marrow-derived dendritic cells
BMMCs: Bone marrow-derived macrophage cells
EXs: Exosomes
DEXs: Dendritic cell-derived exosomes
Gel: Alginate hydrogel
DEXs-Gel: DEXs incorporated with alginate hydrogel
Treg cells: Regulatory T cells
ECM: Extracellular matrix
MSC: Bone marrow mesenchymal stem cell
sEVs: MSC-derived exosomes
TEM: Transmission electron microscopy
NTA: Nanoparticle tracking analysis
SEM: Scanning electron microscopy
