# Correction to: Hydrogel-load exosomes derived from dendritic cells improve cardiac function via Treg cells and the polarization of macrophages following myocardial infarction

**DOI:** 10.1186/s12951-021-01043-8

**Published:** 2021-09-28

**Authors:** Youming Zhang, Zichun Cai, Yunli Shen, Qizheng Lu, Wei Gao, Xin Zhong, Kang Yao, Jie Yuan, Haibo Liu

**Affiliations:** 1 Department of Cardiology, QingPu Branch of Zhongshan Hospital, Fudan University, Shanghai, 201700 People’s Republic of China; 2 Department of Cardiology, Shanghai East Hospital, School of Medicine, Tongji University, Shanghai, 200092 People’s Republic of China; 3 Department of Cardiology, Shanghai East Hospital of Clinical Medical College, Nanjing Medical University, Nanjing, 211166 People’s Republic of China; 4 Shanghai Institute of Cardiovascular Diseases, Zhongshan Hospital, Fudan University, Shanghai, 200032 People’s Republic of China

## Correction to: J Nanobiotechnol 19:271 (2021) https://doi.org/10.1186/s12951-021-01016-x

Following publication of the original article [1], the authors identified some mistakes about the affiliations of the authors. The updated “the affiliations of Authors” is given below and the revisions have been highlighted in bold typeface. The affiliation of the first author "**Youming Zhang**" and the authors "Yunli Shen, Qizheng Lu," should be **Affiliation 2 "Department of Cardiology, Shanghai East Hospital, School of Medicine, Tongji University, Shanghai 200092, People’s Republic of China."** And the affiliation of the author "**Zichun Cai**" should be **Affiliation 3 "Department of Cardiology, Shanghai East Hospital of Clinical Medical College, Nanjing Medical University, Nanjing 211166, People’s Republic of China."**

The original article has been revised.
